# Organic-inorganic hybrid electrochromic materials, polysilsesquioxanes containing triarylamine, changing color from colorless to blue

**DOI:** 10.1038/s41598-017-15337-1

**Published:** 2017-11-07

**Authors:** Shuzhong Wang, Shuwei Cai, Wanan Cai, Haijun Niu, Cheng Wang, Xuduo Bai, Wen Wang, Yanjun Hou

**Affiliations:** 1 Key Laboratory of Functional Inorganic Material Chemistry, Ministry of Education, Department of Macromolecular Science and Engineering, School of Chemical, Chemical Engineering and Materials, Heilongjiang University, Harbin, 150086 P.R. China; 2 School of Materials Science and Engineering, Harbin Institute of Technology, Harbin, 150080 P.R. China

## Abstract

Four kinds of soluble monomers, containing triarylamine (TAA) group with reactive siloxane group, were synthesized under mild conditions via the reaction between 3-(triethoxysilyl)propyl isocyanate (TEOSPIC) and four TAA derivatives, respectively. Then the corresponding colorless organic-inorganic hybrid materials (PSSOs) were derived from the hydrolytic condensation of the monomers. PSSOs revealed good solubility in polar solvents on account of the effect of propeller-like TAA unit as well as the auxo-action effect of the flexible chain within the monomers. The structural characteristics of these PSSOs were identified by ^1^H NMR, ^29^Si NMR, FT-IR spectroscopies and X-ray diffraction (XRD). The morphology, dynamic changes of the transmittance and current before and after electro-oxidizing reaction were studied, and didn’t show significant change suggesting good stability of the PSSOs. Meanwhile, these PSSOs performed high contrast of optical transmittance change up to 84% with the highest coloration efficiency to 241 cm^2^·C^−1^. Furthermore, electrofluorescent properties of PSSOs were investigated with high-contrast.

## Introduction

Electrochromic materials are the materials whose optical properties including the color, transparency *et al*. change with electric charge, which have been widely used in displays, adjustable mirrors, smart windows, information storage and so on^1–8^. Since the first electrochromic material of WO_3_ was reported by Deb, the family of electrochromic materials has become more and more abundant. Electrochromic materials are made up of four distinct categories including metal oxides, coordination complexes, small organic molecule, side-chain-substituted polymers or main-chain polymers^9,10^. Among the electrochromic polymers, TAA and its derivatives are well-known materials due to their excellent properties such that easier film process, multicolor change, high contrast and rapid response and have gained great interest of scientists^11^. Liou, Hsiao, Liaw and other researchers have reported a large number of TAA-based electrochromic materials^12–17^. According to the previous study, the propeller-like TAA unit can play an important role in reducing intermolecular force, which is in favor of improving the solubility of polymers as well as increasing the photoelectric properties. But the effect of improving the solubility of polymers would be weaken if the TAA derivatives contain carbazole, phenothiazine or similar planar structures. So that, introducing a flexible chain in TAA group would be a better way to improve the solubility of such polymers.

In addition, organic conjugated polymers, such as polycarbazole, polypyrrole, polyaniline and polythiophene, have wide application in electrochromic device because of their better stability, faster switching speed, high color contrast rations, compared to inorganic and small organic compounds^18^. But, due to the conjugation structure, the polymers always show deep color in natural state which will deter the application of conductive polymers in architecture window. To get colorless electrochromic materials in natural state, we have researched the electrochromic properties of many polyimides (PIs) containing TAA group, which demonstrated that PIs had good electrochromic performance^19^. However, because of charge transfer complex (CTC) formed among inner- and inter- chain in the main chain, the PI also reveals light brown color. Therefore, we must look for new strategy to obtain colorless host material to meet the requirement.

Fortunately, thanks to polysilsesquioxane (PSSO), a transparent polymer, which owns good optical property, good solubility in common organic solvents, superior film-forming ability, credible adhesion to various substrates and excellent resistance to thermal, chemical and irradiation degradation^20,21^ that render it be particularly attractive for use in a variety of optoelectronic applications. At the same time, special alternate structure of silica oxygen atom reduces the degree of conjugation within polymers. Inspired by this, we believe that the development of easily synthesized PSSO with TAA functional group could be one of the most promising pathways to achieve ideal hosts. PSSO is generally prepared from molecular monomers using hydrolytic condensation of a trialkoxysilane, RSi(OR)_3_. As we know, there are three types of configurations for PSSO: cage, ladderlike, random network structures, which accord with the formulas T_n_(OH)_x_(OR’)_y_ [T = RSiO_1.5−(x+y)/2n_; n = 4,5,6,…; x,y = 0,1,2,…]^22^. Kowalewska has proved the propensity of the hydrolytic condensation process to form linear oligomers at low temperatures, with high amount of H_2_O and at increased time of condensation under acidic conditions. Branched species, random network structure, are more easily formed at higher temperatures^23^. Ren has synthesized a kind of TAA-based PSSO with an *E*_*T*_ (*E*_*g*_ of triplet energy level) of 2.84 eV and a HOMO level of −6.09 eV. as well as improved film-forming ability, thermal, electrochemical, and morphological stability^24^. Jang-Joo Kim synthesized TPD-based crosslinked PSSO containing excellent solvent resistance and electrochemical stability^25^. Zhang prepared three-dimensional organic/inorganic hybrid materials (γ-Glycidoxypropyl)silsesquioxanes), from the hydrolytic condensation of γ-glycidoxypropyl)trimethoxysilane in the presence of an acid catalyst (HCOOH)^22^. Kumaraswamy has prepared hybrid nanomaterials that have a layer thickness of around 1 nm by the condensation of organotrialkoxy silanes^26^.

Herein, four monomers, containing TAA functional group linked with flexible chain and reactive siloxane group, were synthesized through the reaction between amidogen and isocyanato. The corresponding hybrid materials PSSOs were synthesized by the hydrolytic condensation of the monomers. The morphologies of films before and after color change as well as dynamic changes of the transmittance and current were observed and showed no significant change suggesting excellent stability of PSSOs. And the thermal properties and solubility, optical property and electrochemical properties of PSSOs were also investigated.

## Results and Discussion

### Basic characteristics of monomer and PSSOs

**Figure 1:**
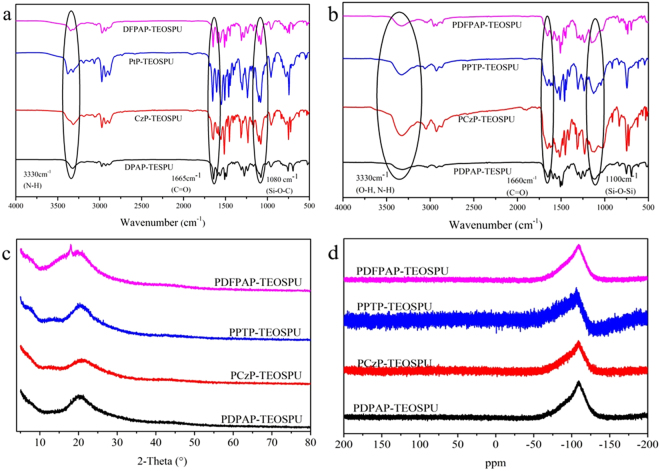
The FT-IR spectra of monomers (**a**) and PSSOs (**b**), XRD patterns (**c**) and ^29^Si NMR spectra (**d**) of PSSOs.

To obtain a pure compound of high yield in the next step, the compounds needed to be purified by recrystallization and column chromatography. The structures of monomers were verified by ^1^H NMR and FT-IR spectrometer which shown in Figure S1–S8 and Fig. 1a. For the synthesis of monomers, the bands at around 3330 cm^−1^, 1660 cm^−1^ and 1080 cm^−1^are assigned to the absorption peaks of N-H, C=O and Si-O-C group, respectively. And the ^1^H-NMR spectra of monomers reveal two peaks at around 6.2 ppm and 8.6 ppm attributed to the proton resonance of the urea group which is consistent with the FT-IR spectra. As can be seen in Fig. 1a and b, the bands at around 1100 cm^−1^ turn sharp into wide peaks after hydrolytic condensation which can be answered by the generation of Si-O-Si group.

Molecular weights and polydispersity (PDI) of PSSOs were measured by GPC using polystyrene standards as standard. The number-average molecular weight $({\overline{M}}_{n})$ of PSSOs are determined to be 9.6 kDa-13.2 kDa. Obviously, the polymerization degrees are greater than 8 which indicate that the principal structure of PSSOs demonstrate random network or ladderlike structure. In order to determine the structure of PSSOs, X-ray diffraction experiments are carried out and the results are shown in Fig. 1c. As shown in Fig. 1c, no sharp peaks were found in the XRD patterns reveals the essentially amorphous structures of PSSOs. In addition, the XRD patterns are in poor agreement with characteristic patterns of ladderlike PSSOs^26–29^ and so that we have reasons to believe that we have obtained random network PSSOs. In order to exclude the almost impossible linear structure of PSSOs, ^29^Si NMR were used to further prove the net structure of PSSOs. As shown in Fig. 1d, asymmetric peaks appeared at about −108 ppm, which indicate that the main form of the Si-atom is –SiO_3/2_. Moreover, terminal molecules, which form into the types of –Si(OH)O_2/2_ and –Si(OH)_2_O_1/2_, may be account for the asymmetry of the peaks.

### Thermal properties, solubility and film morphology

**Figure 2:**
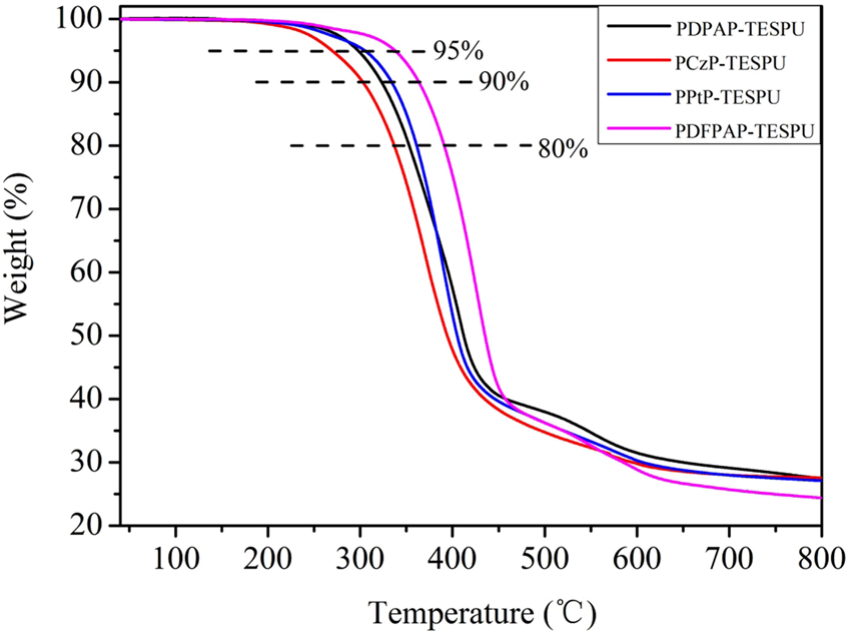
TGA traces of PSSOs.

The thermal behaviors of PSSOs were determined by TGA (shown in Fig. 2). The decomposition temperatures (T_d_) at 5%, 10% and 20% weight losses in nitrogen are summarized in Table 1. The 5% weight loss temperatures of PSSOs in nitrogen were recorded in the range of 270–340 °C, which could meet the requirements of the application. Compared with the polyimides Chang had synthesized^30^, T_d_s of PSSOs are lower which can be attributed to the alkyl chain and the unreacted -OH group in PSSOs backbone. The amount of carbonized residues (char yield) at 800 °C in nitrogen for all PSSOs were in the range of 25–28 wt%. The little char yield can be answered by the high content of -CH_2_-, -OH within PSSOs backbone as we discussed before.

**Table 1:** Thermal properties of PSSOs.

PSSOs	T_5%_^a^	T_10%_^a^	T_20%_^a^	Char Yield^b^(wt%)
PDPAP-TEOSPU	297	323	353	28
PCzP-TEOSPU	270	303	336	28
PPTP-TEOSPU	308	335	361	28
PDFPAP-TEOSPU	340	365	391	25

^a^The decomposition temperatures (T_d_) at 5%, 10% and 20% weight losses in nitrogen.

^b^Measured at 800 °C in nitrogen.

The solubility of PSSOs were measured with a 5 mg sample in 1 mL of a solvent and the results were shown in Table 2. As summarized in Table 2, all PSSOs have good solubility in polar solvents such as DMF, DMSO, N,N-dimethylacetamide (DMAc), m-Cresol and N-methyl pyrrolidone (NMP). The enhanced solubility can be attributed to the introduction of the propeller-like TAA group and the influence of flexible chain which decreases inter-chain interaction and increases the free volume. Thus, these PSSOs could have a wide application though solution processing.

**Table 2:** Solubility of PSSOs in common organic solvent.

PSSOs	Solubility in various Solvent^a^								
	NMP	DMAc	DMF	DMSO	*m*-cresol	THF	Toluene	CH_3_CN	n-hexane
PDPAP-TEOSPU	++	++	++	++	++	+	+−	—	—
PCzP-TEOSPU	++	++	++	++	++	+	+−	—	—
PPTP-TEOSPU	++	++	++	++	++	+	+−	—	—
PDFPAP-TEOSPU	++	++	++	++	++	+	+−	—	—

^a^The solubility were measured with a 5 mg sample in 1 mL of a solvent. ++, soluble at room temperature; +, soluble on heating; +−, partially soluble or swelling; −, insoluble even on heating.

**Figure 3:**
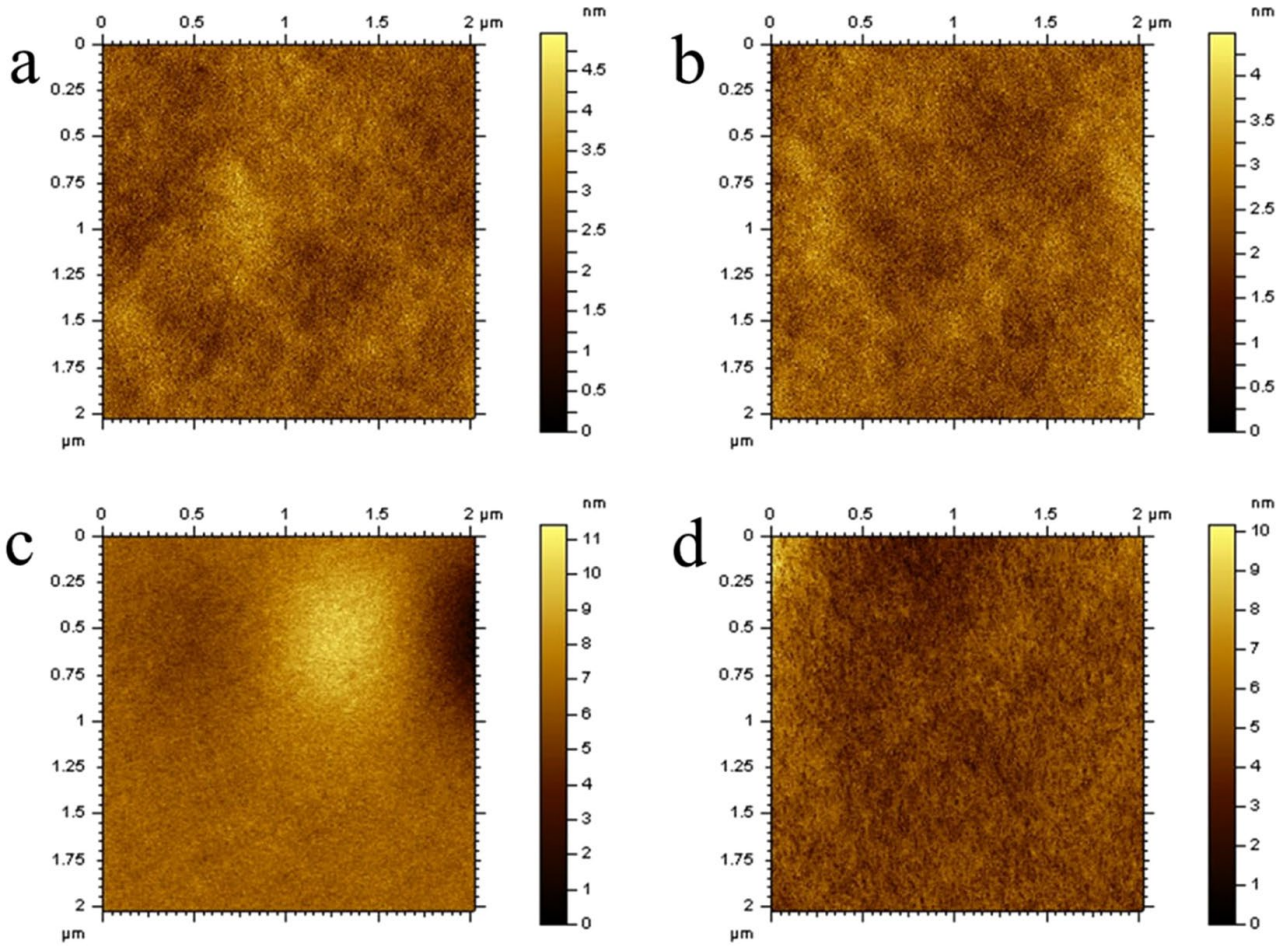
AFM images of (**a**), (**b**) for PDFPAP-TEOSPU; (**c**), (**d**) for PCzP-TEOSPU before and after CV experiment in tapping mode.

The tapping-mode AFM topographical images (2 μm × 2 μm) of PSSO films are shown in Fig. 3 to estimate the morphology evolution in a microstructure and stability before and after oxidation process. Figure 3a,b reveal the morphology of PDFPAP-TEOSPU film before and after oxidation and Fig. 3c,d represent that of PCzP-TEOSPU film. The AFM topographical images of PDPAP-TEOSPU and PPTP-TEOSPU are shown in Figure S10. All the images show no particular agglomeration phenomenon and breakage for all the PSSO films. The morphology of the PSSO films does not change significantly in appearance after CV process as shown in Fig. 3. This means that PSSO films possess good stability in progress of CV test which point to excellent electrochromic reversibility. Roughness of films before oxidation process are determined to be 7.83 nm for PDPAP-TEOSPU, 0.598 nm for PCzP-TEOSPU, 1.16 nm for PPTP-TEOSPU and 0.979 nm for PDFPAP-TEOSPU, respectively. Low roughness shows a smooth film on account of the PSSOs’ superior film-forming ability with ITO substrate as well as good wetting capacity of DMF to ITO substrate.

### Optical property

**Figure 4:**
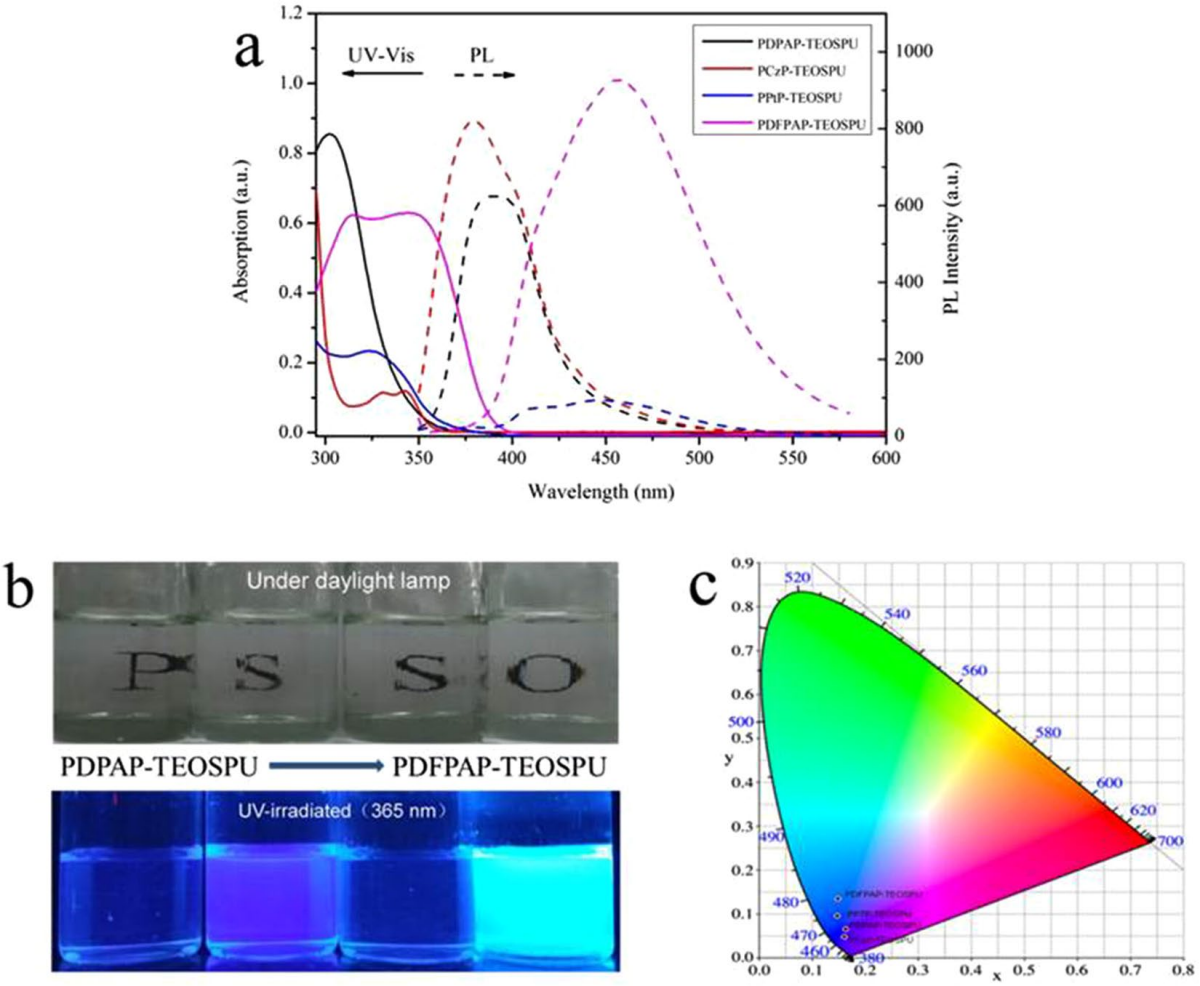
(**a**) UV-vis absorption and PL spectra of PSSOs, (**b**) Photographs of PSSOs in DMF solution under daylight lamp and 365 nm wavelength UV light, (**c**) the CIE 1931(x,y) chromaticity diagram of PSSOs. The “PSSO” letter can be seen behind the solution.

The optical properties of the PSSOs were investigated by the UV-vis and photoluminescence (PL) spectroscopy (Fig. 4), and the results were summarized in Table 3. PSSOs exhibit maximum UV-vis absorption at 302–346 nm in DMF solution, which ascribed to the π-π* transitions between the center nitrogen atoms of the TAA group and benzene ring^31^. When PSSOs were excited at the maximum absorption wavelength, the emission peaks were observed at 380–456 nm, respectively. A bathochromic shift of emission in the PL spectra of PDFPAP-TEOSPU, which caused by the more extended conjugated fluorene group, has been found. UV-vis absorption follows a similar tendency. PL quantum yields *φ*s of PSSOs were measured by using quinine sulfate dissolved in 0.5 mol·L^−1^ sulfuric acids as reference standard (*φ* = 0.546). The *φ*s of PSSOs after refractive index correction were calculated according to the following formula^32^

$${\varphi }_{unk}={\varphi }_{std}(\frac{{I}_{unk}}{{I}_{std}})(\frac{{A}_{std}}{{A}_{unk}}){(\frac{{\eta }_{unk}}{{\eta }_{std}})}^{2}$$

where *φ*_unk_, *φ*_std_, I_unk_, I_std_, A_unk_, A_std_, *η*_unk_, and *η*_std_ are the fluorescent quantum yield, integration of the emission intensity, absorbance at the excitation wavelength, and the refractive indices of the corresponding solutions for the samples and the standard, respectively. Here, we use the refractive indices of the pure solvents as those of the solutions. PSSOs in DMF solution exhibited fluorescence emission maxima at 380–456 nm with quantum yields ranging from 1.23% for PDPAP-TEOSPU to 39.05% for PDFPAP-TEOSPU. PCzP-TEOSPU and PDFPAP-TEOSPU exhibit higher fluorescence quantum yields due to the presence of bulky, rigid carbazole and fluorene segments in the network. The low fluorescence of PDPAP-TEOSPU can be attributed to the TAA-containing structure, with the greater conformational mobility resulting in an increased nonradiative decay^33^. For PSSOs, their emission colors range from purplish blue (PCzP-TEOSPU, CIE 1931: x, 0.1606; y, 0.0485) to blue (PDFPAP-TEOSPU, CIE 1931: x, 0.1476; y, 0.1344), respectively.

**Table 3:** Optical Properties of PSSOs.

PSSOs	In Solution^a^			As film	
	${{\boldsymbol{\lambda }}}_{{\bf{\max }}}^{{\bf{abs}}}$(nm)	${{\boldsymbol{\lambda }}}_{{\bf{\max }}}^{{\bf{PL}}}$(nm)	${\varphi }_{{\rm{F}}}$(%)^b^	${{\boldsymbol{\lambda }}}_{{\bf{\max }}}^{{\bf{abs}}}$(nm)	${{\boldsymbol{\lambda }}}_{{\bf{onset}}}^{{\bf{abs}}}$(nm)
PDPAP-TEOSPU	302	389	1.23	320	388
PCzP-TEOSPU	335	380	26.15	341	390
PPTP-TEOSPU	327	446	1.12	332	372
PDFPAP-TEOSPU	346	456	39.05	357	399

^a^Measured in dilute solutions in DMF at a concentration of about 10^−5^ mol·L^−1^.

^b^Fluorescence quantum yield calculated with quinine sulfate as the standard reference. (*ϕ*_*F*_ = 54.6%).

### Electrochemistry properties

**Figure 5:**
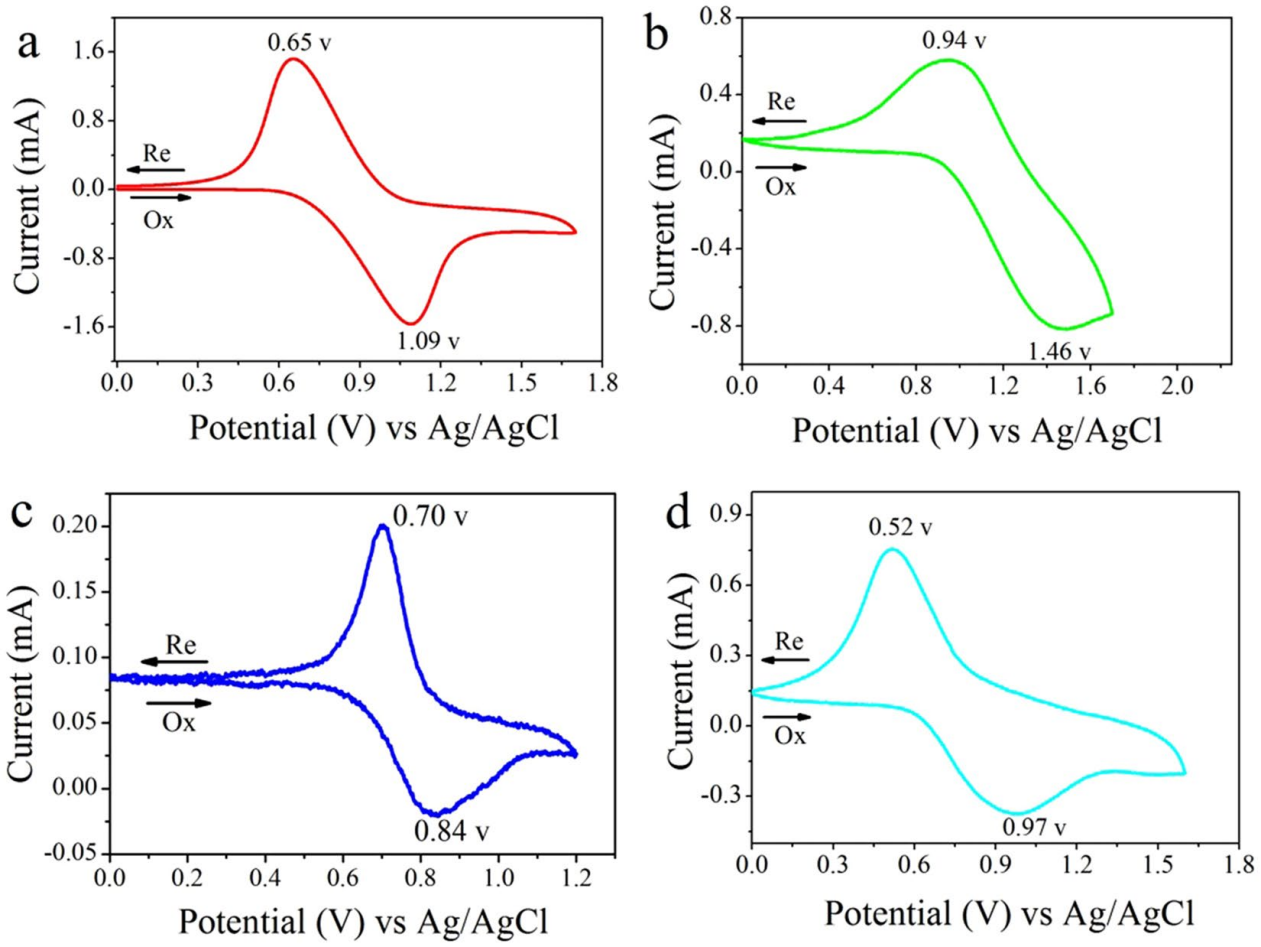
Cyclic voltammograms of PDPAP-TEOSPU (**a**), PCzP-TEOSPU (**b**), PPTP-TEOSPU(**c**), PDFPAP-TEOSPU (**d**) in 0.1 mol·L^−1^ LiClO_4_/CH_3_CN solution at a scan rate of 50 mV·s^−1^.

**Figure 6:**
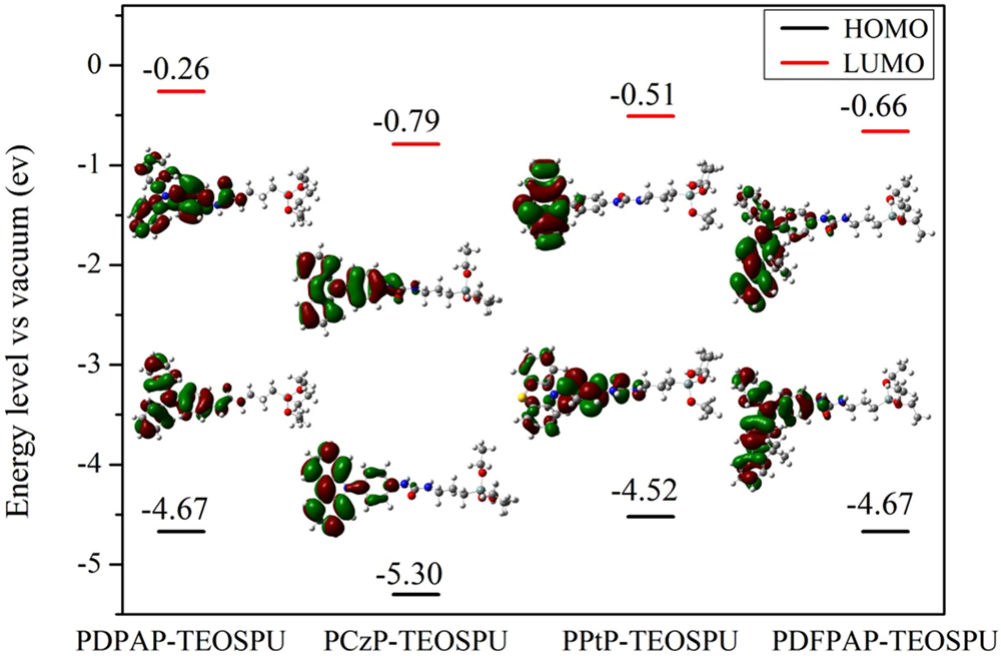
Proposed simplified redox process and the resonance form of PSSOs.

The electrochemical behaviors of PSSOs were investigated by CV technique conducted for the cast film on an ITO-coated glass substrate as working electrode in 0.1 mol·L^−1^ LiClO_4_/CH_3_CN solution. The derived oxidation potentials are summarized in Fig. 5 and Table 4. As can be seen in Fig. 5, a reversible redox process with a pair of redox peaks are observed for each PSSO, which are attributable to the oxidation of N atoms in the TAA groups. As an example, the graphs of PDPAP-TEOSPU exhibited a oxidation peaks at 1.09 V and corresponding reduction peak at 0.65 V, which can be ascribed to the formation of TAA ↔ TAA^+•^, respectively. Obviously, oxidation potential of PPTP-TEOSPU is lower than that of other PSSOs. The phenomenon may be caused by the stronger electron-donor of phenothiazin which gives electrons and results that the linked N atom is easier to be oxidized. On the other hand, for PCzP-TEOSPU, a huger ringlike π conjugated structure was formed between the lone pair electros on N atom and π electron of benzene ring in carbazole group, therefore it is difficult for the electrons to leave. Therefore, PCzP-TEOSPU owns the highest oxidation potential. And when the potential was applied, the polymer films exhibited an obvious color change from colorless to dark blue for PDPAP-TEOSPU, blue for PCzP-TEOSPU, red for PPTP-TEOSPU and cyan for PFPAP-TEOSPU which can be seen in the inset pictures of Figures S11a, S12a and S13a. According to these results, proposed mechanisms of oxidation reactions for PSSOs are exhibited in Fig. 6.

**Table 4:** Electron density in the frontier molecular orbitals of monomers and *E*_g_.

PSSOs	${{\boldsymbol{\lambda }}}_{{\boldsymbol{onset}}}^{{\boldsymbol{abs}}}$ ^a^	${{\boldsymbol{E}}}_{{\boldsymbol{onset}}}^{{\boldsymbol{peak}}}$ ^**b**^_**vs Ag/AgCl**_	${{\boldsymbol{E}}}_{{\boldsymbol{HOMO}}}^{{\boldsymbol{electro}}}$ ^**c**^	${{\boldsymbol{E}}}_{{\boldsymbol{LUMO}}}^{{\boldsymbol{electro}}}$ ^**c**^	${{\boldsymbol{E}}}_{{\boldsymbol{g}}}$ ^**d**^	${{\boldsymbol{E}}}_{{\boldsymbol{HOMO}}}^{{\boldsymbol{quntum}}}$ ^**e**^	${{\boldsymbol{E}}}_{{\boldsymbol{LUMOH}}}^{{\boldsymbol{quntum}}}$ ^**e**^	${{\boldsymbol{E}}}_{{\boldsymbol{g}}}^{{\boldsymbol{quntum}}}$ ^**e**^
PDPAP-TEOSPU	388	0.68	−5.03	−1.83	3.20	−4.67	−0.26	4.41
PCzP-TEOSPU	362	0.85	−5.20	−1.92	3.28	−5.30	−0.79	4.51
PPTP-TEOSPU	372	0.62	−4.97	−1.64	3.33	−4.52	−0.51	4.01
PDFPAP-TEOSPU	468	0.63	−4.98	−1.87	3.11	−4.67	−0.66	4.01

^a^Onset absorption wavelength of PSSO films.

^b^From CV vs Ag/AgCl in LiClO_4_/CH_3_CN. ${E}_{onset}^{peak}$: Onset potential of PSSOs’ CV curve.

^c^E_HOMO_ = −e ($${E}_{onset}^{peak}$$_vs Ag/AgCl_ + 4.35) V; E_LUMO_ = E_HOMO_ + E_g_.

^d^ *E*_g_ = 1240/λ_onset_.

^e^Theoretical calculation of PSSOs.

### Quantum chemistry calculation

**Figure 7:**
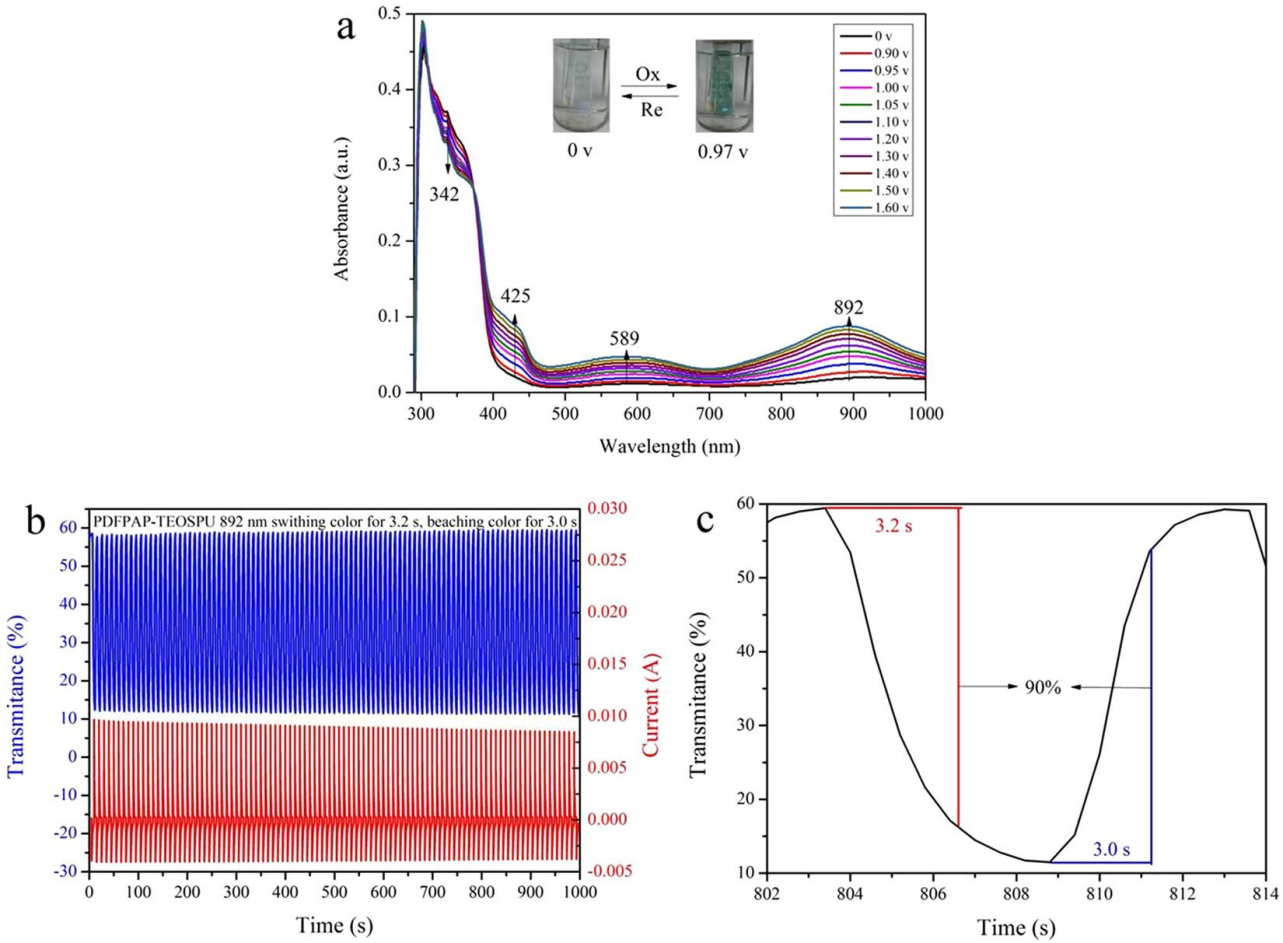
Pictorial representations of the electron cloud in the frontier molecular orbitals of monomers.

The DFT theory is used to better understand the evolution of redox potentials and electronic structure of the monomers. Figure 7 shows the electron cloud distribution in HOMO and LUMO state. The electrons of HOMO and LUMO state are both localized at the TAA unite. This implies that charge transfer between electron-donor and electro-acceptor moieties, which occurs in the process of the transition from ground state to excited state, is within the TAA unite. In the monomers, the theoretical trend of *E*_*g*_ is in the order of DFPAP-TEOSPU < PTP-TEOSPU < DPAP-TEOSPU < CzP-TEOSPU; and the experimental data are in the order of PDFPAP-TEOSPU < PDPAP-TEOSPU < PCzP-TEOSPU < PPtP-TEOSPU. The slight deviation may be caused by the influence of polymerization, solvent and the force of intermolecular. No matter theoretical or experimental data, the smallest *E*_*g*_ of PDFPAP-TEOSPU is due to the more extended conjugation fluorene group. Moreover, theoretical data were only resulted from the monomers, not from whole PSSOs. Meanwhile, the solvent and electrolyte also play an important effect on the result.

### Electrochromic properties of PSSOs

**Figure 8:**
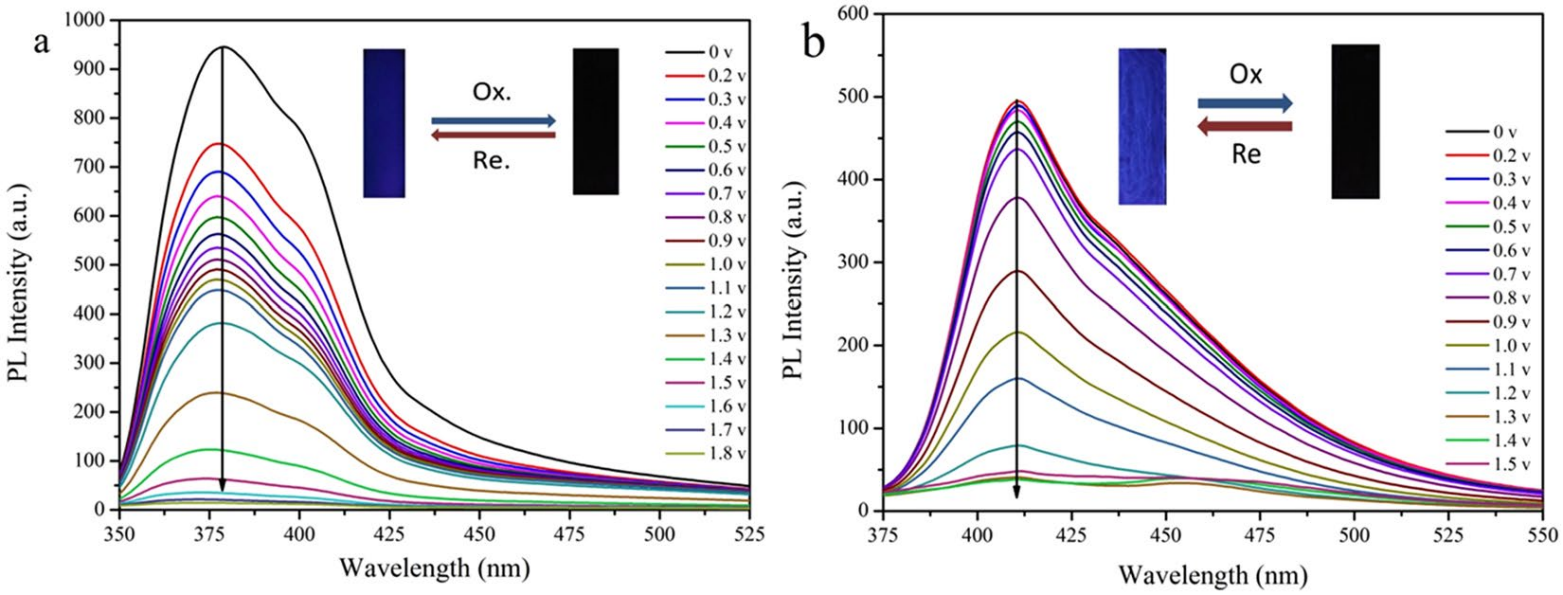
Electronic absorption spectra of PDFPAP-TEOSPU thin film (**a**), dynamic changes of the transmittance and current (**b**) and optical switching (c) for PDFPAP-TEOSPU (in 0.1 mol·L^−1^ LiClO_4_/CH_3_CN solution) by applying a step potential.

As shown in Fig. 8a, PDFPAP-TEOSPU film exhibits strong absorption at 305 nm at 0 V due to π-π* transitions of TAA and fluorene group, and there is no significant absorption in the visible light region. The absorption of the film is gradually weakened at 342 nm, while new absorptions emerge at 425, 589 and 892 nm in NIR region with voltage increasing from 0 to 1.6 V. It is believed that it is the characteristic result of the oxidation process when a monocation radical (TAA^+•^) was formed. The color changes from colorless to cyan, which can be seen in the inset pictures of Fig. 8a. The color changes of other PSSOs are shown in Figures S11a, S12a and S13a.

The stability of PDFPAP-TEOSPU film was estimated through potential step absorptiometry between 0–1.5 V (vs Ag/AgCl) at 892 nm. Its current and transmittance basically remained unchanged after 100 cycles with a cycle time of 10 s as displayed in Fig. 8b, which indicated that PDFPAP-TEOSPU possessed excellent electrochromic reversibility in according to the conclusion of morphology study. And the electronic absorption spectra of other PSSOs were shown in Figures S11b, S12b and S13b, which revealed similar electrochromic reversibility.

Response time, one of the most important characteristics of electrochromic materials, is the time needed to perform a switching between the initial and oxidized states of the materials^34^, which is defined as the time required for reach 90% of the full change in absorbance after the switching of the potential. And the related data are summarized in Table 5. As shown in Fig. 8c, the film of PDFPAP-TEOSPU requires 3.2 s for coloration and 3.0 s for bleaching at 892 nm. Fast converting speed can completely satisfy the requirement of practical application.

**Table 5:** Electrochromic properties of PSSOs.

PSSOs	λ (nm)^a^	ΔT (%)	t_s_(s)^b^	t_b_(s)^c^	δ_OD_^d^	Q(mC·cm^−2^)^e^	η(cm^2^·C^−1^)^f^
PDPAP-TEOSPU	774	84	3.1	3.0	1.37	7.300	188
PCzP-TEOSPU	1000	56	3.6	3.6	0.84	3.506	241
PPTP-TEOSPU	520	4	1.8	3.0	0.02	1.496	15
PDFPAP-TEOSPU	892	48	3.2	3.0	0.72	3.718	194

^a^Wavelength of absorption maximum.

^b^Time for swiching color.

^c^Time for beaching color.

^d^Optical density (Δ_OD_) = lg[T_bleached_/T_colored_], where T_colored_ is the maximum transmittance in the oxidized state and that of T_bleached_ is in the neutral state.

^e^Q_d_ is ejected charge.

^f^Coloration efficiency (CE) η = Δ_OD_/Q_d_.

The electrochromic coloration efficiency (CE, η) is also an important characteristic for the electrochromic materials. CE can be calculated using the equations and given below^35^:

$$\begin{array}{c}{{\rm{\Delta }}}_{{\rm{OD}}}=\,{\rm{lg}}({{\rm{T}}}_{{\rm{b}}}/{{\rm{T}}}_{{\rm{c}}})\\ {\rm{\eta }}={{\rm{\Delta }}}_{{\rm{OD}}}/{\rm{Q}}\end{array}$$

where T_b_ and T_c_ denote the transmittances of the film before and after colorations, respectively. Δ_OD_ is the change of the optical density, which is proportional to the amount of created color centers. η denotes the coloration efficiency (CE). Q (mC·cm^−2^) is the amount of injected charge per unit sample area. As shown in Table 5, PDPAP-TEOSPU, PCzP-TEOSPU, PDFPAP-TEOSPU owns higher CE values above 180 cm^2^·C^−1^ and PPTP-TEOSPU owned the lowest CE due to its high transmittance at oxidized states shown in Figure S13.

### Electrofluorescent properties

**Figure 9:**
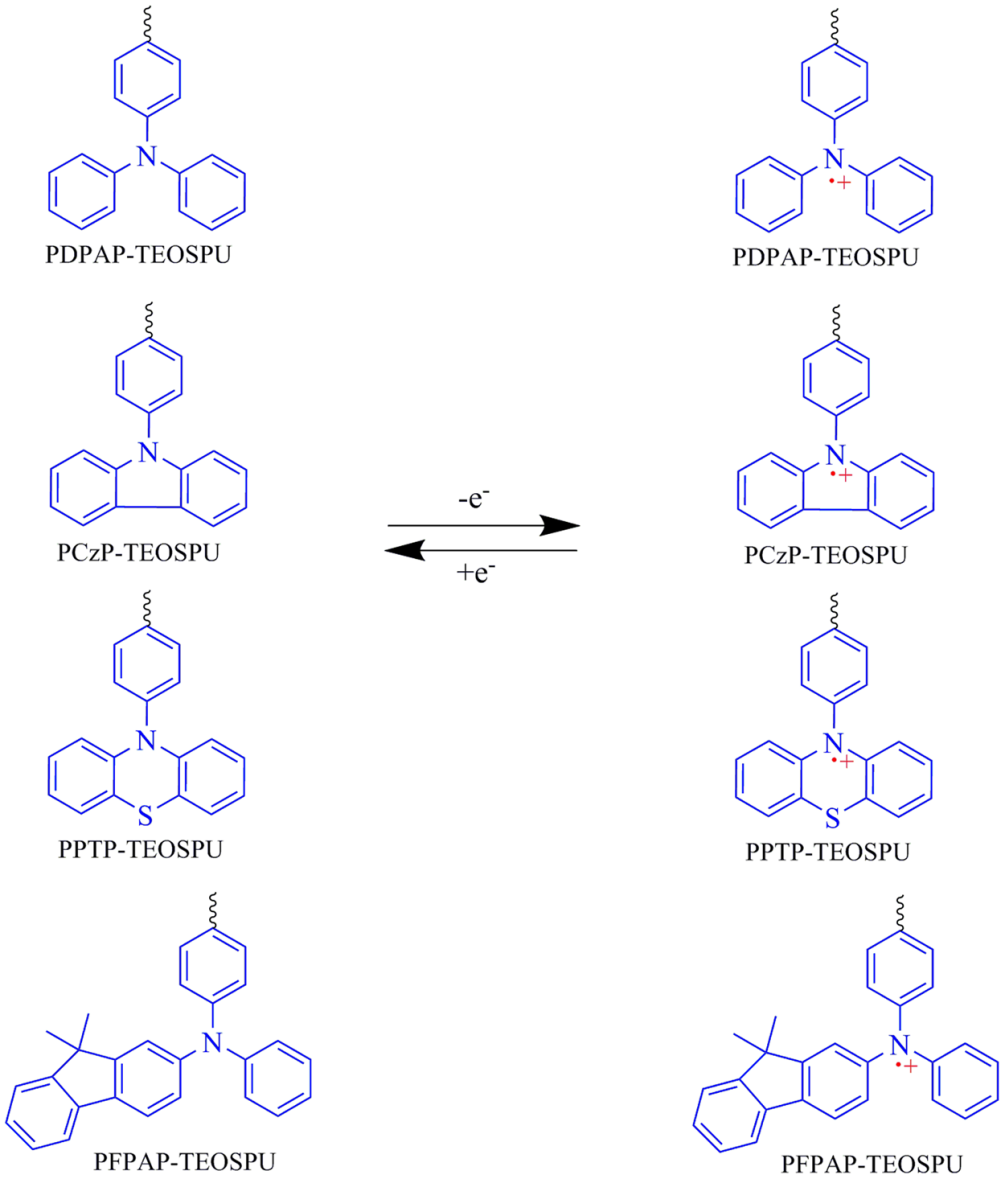
Fluorescence changes of PCzP-TEOSPU (**a**) and PDFPAP-TEOSPU (**b**) at different applied potentials. The inset images shows the fluorescence changes under UV excitation (365 nm).

Electrofluorescent measurements were carried out to evaluate the optical properties in the ways of Sun has reported^36^. As we know, TAA^+•^ is an effective fluorescence quencher^37^ and thus the fluorescence of PSSOs containing TAA could be readily controlled by voltage. On basis of the strong fluorescence of the initial PSSOs, the emission spectra of PCzP-TEOSPU and PDFPAP- TEOSPU under different applied potential were collected. As shown in Fig. 9, when PSSOs were excited at the maximum absorption wavelength (shown in Table 3), the emission peaks were observed at 378 nm for PCzP-TEOSPU and 411 nm for PDFPAP-TEOSPU, respectively. Obviously, the emission peaks of films revealed hypsochromic shift comparing that of corresponding solution by the reason of aggregation effect. Furthermore, no shift of spectra band with voltage increasing suggested that no side-reactions occur during the oxidation process from TPA to TAA^+• ^^38^. Reversibility of the electrofluorescence were also studied and were shown in Figure S14. Contrary to our will, reversibility of the two PSSOs were not as good as we expected. After 10 times, the intensity of fluorescence can’t be restored to initial state. Decay in air and the destroy of ultraviolet light may be responsible for this matter. To prove the conjecture, we measured the emission spectra of PCzP-TEOSPU and PDFDAP-TEOSPU films in the same measurement setup but no potential applied. As shown in Figure S15, PL intensity of PCzP-TEOSPU and PDFPAP-TEOSPU films reveal distinct weak decay as time goes on. So, we can conclude that decay in air and destroy of ultraviolet light may play a very important role in the poor reversibility of electrofluorescence. Moreover, current damage to PSSOs structure also may be a reason of the poor reversibility.

## Conclusion

Four kinds of soluble monomers were synthesized under mild conditions via the classic organic reaction. The corresponding hybrid materials PSSOs were obtained by the hydrolytic condensation of mono mers. ^1^H NMR, ^29^Si NMR, FT-IR and XRD results turn out that we have synthesized network hybrid materials successfully. All PSSOs reveal good solubility in polar solvents. At the same time, colorless PSSO films in natural state were obtained through introducing TAA group into PSSO backbones. TGA traces reveal good thermal resistance which could meet the requirement of the application. The morphologies of PSSOs after electro-oxidizing reaction, dynamic changes of the transmittance and current were studied and did not reveal significant change suggesting excellent stability of PSSOs. One reversible pair of distinct redox peaks associated with noticeable color changed from colorless to blue, red and cyan for polymer films could be observed in the CV test. Meanwhile, PSSOs performed high contrast of optical transmittance change up to 84% with the highest coloration efficiency up to 241 cm^2^·C^−1^. Furthermore, the electrofluorescent properties of PCzP-TEOSPU and PDFPAP-TEOSPU were also investigated here and reveal high-contrast. All the results suggest that we have synthesized good electrochromic materials via introducing TAA group into PSSO backbone.

## Methods

### Materials

Diphenylamine, carbazole, phenothiazine, 9,9-dimethyl-N-phenyl-9H-fluoren-2 –amine were purchased from TCI Co. 80% hydrazine hydrate and 3-(triethoxysilyl)propyl isocyana (TEOSPIC)were purchased from Aldrich Co. Other materials were bought from Sinopharm Chemical Reagent Ltd., Co., China. Sodium hydride and N,N-dimethylformamide (DMF) was used as received. Dimethyl sulfoxide (DMSO) was dried and distilled over calcium hydride before used Tetrahydrofuran (THF) was dried and distilled over Na.

### Synthesis of TAA derivatives

The TAA and derivatives were prepared according to the literature^39^. The synthetic routes are shown in Figure S16.

### Synthesis of monomers

**Figure 10:**
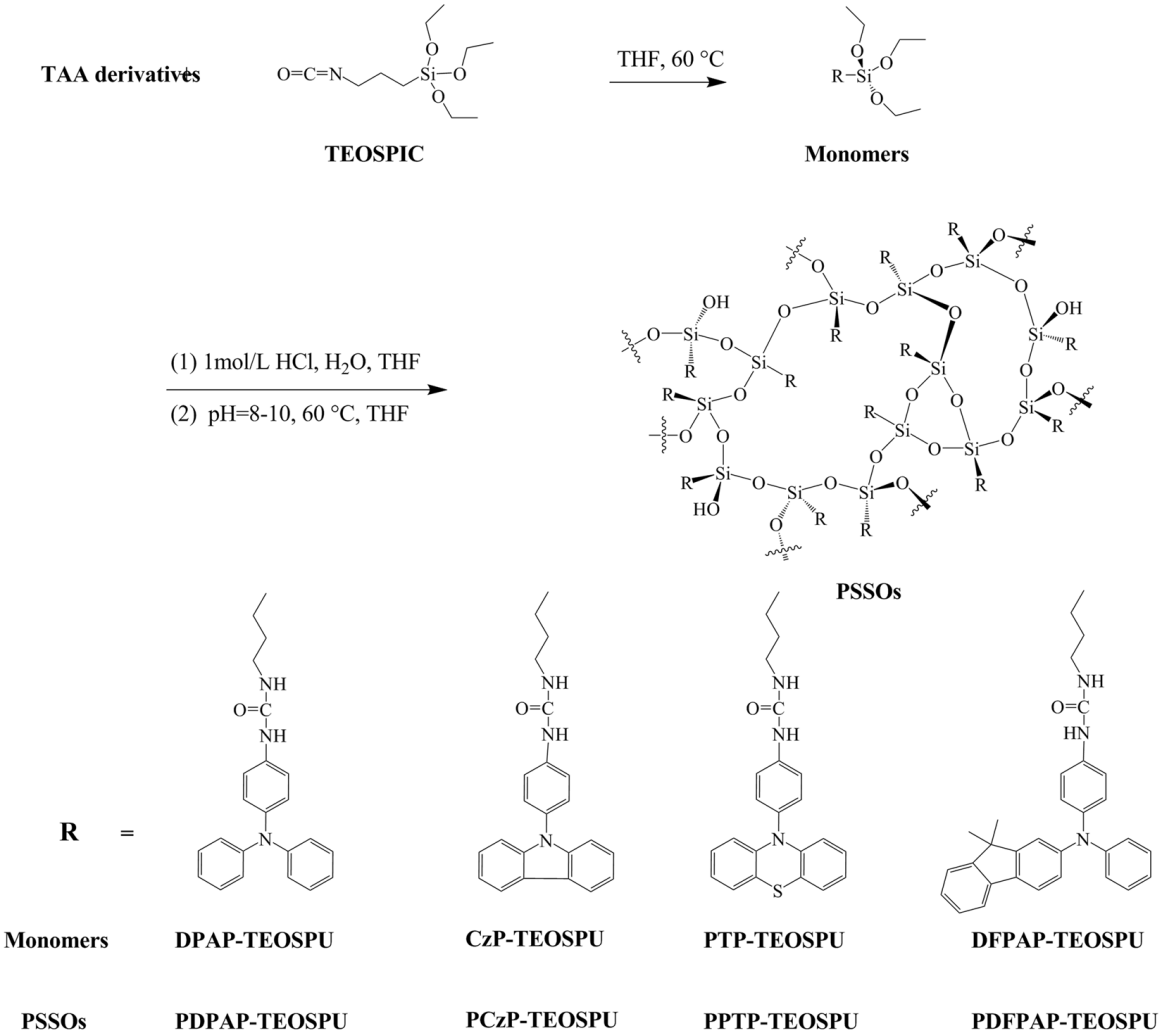
Synthesis routes of PSSOs.

The synthesis of 1-(4-(diphenylamino)phenyl)-3-(3-(triethoxysilyl) propyl)urea (DPAP-TEOSPU), shown in Fig. 10, was used as an example to illustrate the general synthetic procedure. Under N_2_ atmosphere, 2.60 g (0.01 mol) An-TAA was dissolved in dried THF with magnetic stirring at 60 °C and 2.72 g (0.011 mol) TEOSPIC was dropped in within an hour. The reaction was continued for 4 h and then precipitated in ether, dried in vacuum at 40 °C for 5 h. The mixture was purified by column chromatography on silica gel eluting with ethyl acetate/dichloromethane (1/8, V/V) to give a white solid (3.32 g, 65%).

The spectral characterizations of the four monomers are as follows:

1-(4-(diphenylamino)phenyl)-3-(3-(triethoxysilyl)propyl)urea (DPAP-TEOSPU) IR (KBr, cm^−1^): v = 3320 (N-H), 2970–2884 (CH_2_CH_3_), 1640 (C=O), 1598, 1493 (aromatic ring of benzene), 1084 (Si-O-C). ^1^H NMR (400 MHz, DMSO-*d*_6_, ppm): δ = 0.53–0.57 (t, 2); 1.13–1.16 (t, 9); 1.43–1.51 (quint, 2); 3.02–3.06 (q, 2); 3.72–3.77 (q, 6); 6.10–6.13 (t, 1); 6.92–6.97 (m, 6); 7.22–7.26 (t, 2); 7.34–7.36 (d, 2); 8.37 (s, 1).^13^C NMR (100 Hz, DMSO-*d*_6_, δ, ppm): 7.8 (C^c^), 18.7 (C^a^), 23.8 (C^d^), 42.2 (C^e^), 58.2 (C^b^), 119.5 (C^h^), 122.3 (C^n^), 122.8 (C^l^), 126.7 (C^i^), 129.7 (C^m^), 137.5 (C^g^), 140.7 (C^j^), 148.1 (C^k^), 155.7 (C^f^). Yield: 65%.

1-(4-(9H-carbazol-9-yl)phenyl)-3-(3-(triethoxysilyl)propyl)urea (CzP-TEOSPU) IR (KBr, cm^−1^): v = 3319 (N-H), 2971–2880 (CH_2_CH_3_), 1649 (C=O), 1597, 1514 (aromatic ring of benzene), 1077 (Si-O-C). ^1^H NMR (400 MHz, DMSO-*d*_6_, ppm): δ = 0.54–0.58 (t, 2); 1.12–1.15 (t, 9); 1.46–1.53 (quint, 2); 3.05–3.10 (q, 2); 3.71–3.76 (q, 6); 6.23–6.26 (t, 1); 7.21–7.29 (quint, 4); 7.37–7.42 (q, 4); 7.63–7.66 (d, 2); 8.67 (s, 1). ^13^C NMR (100 Hz, DMSO-*d*_6_, δ, ppm): 7.8 (C^c^), 18.3 (C^a^), 23.7 (C^d^), 42.8 (C^e^), 58.6 (C^b^), 109.7 (C^l^), 119.8 (C^o^), 120.2 (C^n^), 120.8 (C^i^), 123.2 (C^h^), 125.9 (C^p^), 127.8 (C^m^), 138.6 (C^g^), 141.1 (C^k^), 156.4 (C^f^). Yield: 62%.

1-(4-(10H-phenothiazin-10-yl)phenyl)-3-(3-(triethoxysilyl)propyl)urea (PTP-TEOSPU) IR (KBr, cm^−1^): v = 3376 (N-H), 2972–2880 (CH_2_CH_3_), 1655 (C=O), 1605, 1510 (aromatic ring of benzene), 1088 (Si-O-C). ^1^H NMR (400 MHz, DMSO-*d*_6_, ppm): δ = 0.53–0.58 (t, 2); 1.12–1.15 (t, 9); 1.45–1.53 (quint, 2); 3.04–3.10 (q, 2); 3.71–3.76 (q, 6); 6.13–6.15 (d, 2); 6.23–6.26 (t, 1); 6.77–6.80 (t, 2); 6.85–6.89 (t, 2); 6.98–7.00(d, 2); 7.21–7.23 (d, 2); 7.63–7.65 (d, 2); 8.66 (s, 1). ^13^C NMR (100 Hz, DMSO-*d*_6_, δ, ppm): 7.8 (C^c^), 18.7 (C^a^), 23.8 (C^d^), 42.3 (C^e^), 58.2 (C^b^), 116.0 (C^l^), 119.2 (C^p^), 120.0 (C^h^), 122.8 (C^n^), 126.9 (C^o^), 127.6 (C^m^), 131.4 (C^i^), 133.1 (C^j^), 141.1 (C^g^), 144.5 (C^k^), 155.6 (C^f^). Yield: 70%.

1-(4-((9,9-dimethyl-9H-fluoren-2-yl)(phenyl)amino)phenyl)-3-(3-(triethoxysilyl)propyl)urea (DFPAP-TEOSPU) IR (KBr, cm^−1^): v = 3342 (N-H), 2975–2887 (CH_2_CH_3_), 1646 (C=O), 1596, 1512 (aromatic ring of benzene), 1077 (Si-O-C). ^1^H NMR (400 MHz, DMSO-*d*_6_, ppm): δ = 0.51–0.55 (t, 2); 1.10–1.13 (t, 9); 1.30 (s, 6); 1.42–1.50 (quint, 2); 3.01–3.06 (q, 2); 3.69–3.76 (q, 6); 6.10–6.13 (t, 1); 6.85–6.87 (dd, 1); 6.90–6.97 (m, 5); 7.10 (d, 1); 7.18–7.27 (m, 3); 7.33–7.36 (d, 2); 7.42–7.44 (d, 1); 7.62–7.66 (t, 2); 8.38 (s, 1). ^13^C NMR (100 Hz, DMSO-*d*_6_, δ, ppm): 7.7 (C^c^), 18.7 (C^a^), 23.8 (C^d^), 27.3 (Co), 42.2 (C^e^), 46.8 (C^n^), 58.2 (C^b^), 117.3 (C^x^), 119.4 (C^i^), 119.8 (C^w^), 121.4 (C^q^), 122.2 (C^l^), 122.3 (C^z^), 122.8 (C^h^), 123.1 (C^r^), 126.5 (C^s^), 126.8 (C^t^), 127.5 (C^b’^), 129.7 (C^a’^), 133.3 (C^g^), 137.4 (C^v^), 138.9 (C^k^), 140.8 (C^u^), 147.6 (C^j^), 148.2 (C^y^), 153.5 (C^p^), 155.1 (C^m^), 155.7 (C^f^). Yield: 67%.

### Synthesis of the PSSOs

As an example, the procedure for PDPAP-TEOSPU is described. A 250 mL round-bottom flask was charged with 0.507 g DPAP-TEOSPU (0.001 mol), 40 mL THF, 100 μL H_2_O and five drops concentrated hydrochloric acid under stirring at room temperature. After 24 h of magnetic stirring, 1 mol·L^−1^ sodium hydroxide was added to neutralize the acidity of the medium and hence increase the condensation reaction rate. At the same time, the temperature was raised to 60 °C and maintained for several days until few floccule appearing. The resulting solution was poured slowly into 400 mL of stirring methanol giving rise to a white precipitate that was collected by filtration, washed thoroughly with methanol, and dried at 45 °C for 15 h in vacuum. The other PSSOs were prepared by an analogous procedure and named as shown in Fig. 10.

PDPAP-TEOSPU: FT-IR (KBr, cm^−1^): v = 3449–3124 (N-H, O-H); 1647(C=O); 1107 (Si-O-Si). ^1^H NMR: (DMSO-*d*_6_, 400 MHz, ppm): 8.31, 6.14 (N-H); 7.40–6.49 (aromatic ring of benzene); 4.43–3.98 (O-H); 3.52–0.34 (CH_2_). ${\overline{M}}_{n}$ = 9.6 kDa, ${\overline{M}}_{w}$ = 13.6 kDa, PDI = 1.42. Yield: 76%.

PCzP-TEOSPU: FT-IR (KBr, cm^−1^): v = 3455–3148 (N-H, O-H); 1663 (C=O); 1123 (Si-O-Si). ^1^H NMR: (DMSO-*d*_6_, 400 MHz, ppm): 8.64, 6.37 (N-H); 8.16–6.52 (aromatic ring of benzene); 4.40–3.98 (O-H); 3.54–0.44 (CH_2_). ${\overline{M}}_{n}$ = 13.3 kDa, ${\overline{M}}_{w}$ = 18.2 kDa, PDI = 1.37. Yield: 82%.

PPTP-TEOSPU: FT-IR (KBr, cm^−1^): v = 3450–3173 (N-H, O-H); 1656 (C=O); 1133 (Si-O-Si). ^1^H NMR: (DMSO-*d*_6_, 400 MHz, ppm): 8.64, 6.00 (N-H); 7.76–6.17 (aromatic ring of benzene); 4.37–3.98 (O-H); 3.52–0.48 (CH_2_). ${\overline{M}}_{n}$ = 11.8 kDa, ${\overline{M}}_{w}$ = 16.6 kDa, PDI = 1.41. Yield: 74%.

PDFPAP-TEOSPU: FT-IR (KBr, cm^−1^): v = 3448–3162 (N-H, O-H); 1654 (C=O); 1156 (Si-O-Si). ^1^H NMR: (DMSO-*d*_6_, 400 MHz, ppm): 8.32, 6.48 (N-H); 7.72–6.48 (aromatic ring of benzene); 4.38–3.96 (O-H); 3.49–0.40 (CH_2_). ${\overline{M}}_{n}$ = 12.4 kDa, ${\overline{M}}_{w}$ = 22.8 kDa, PDI = 1.84. Yield: 86%.

#### Preparation of films for research

A solution of 0.01 g PDPAP-TEOSPU in 1.0 mL DMF was cast on an ITO conductive glass and then placed in a wet atmosphere until the solvent evaporated. The temperature was then raised to 180 °C and kept for 1 h to solidify the films under argon atmosphere.

### Measurements

Fourier transform infrared (FT-IR) spectra were recorded on a PerkinElmer Spectrum 100 Model FT-IR spectrometer. ^1^H NMR and ^13^C NMR and ^29^Si NMR spectra were measured on a Bruker AC-400 MHz spectrometer in CDCl_3_ and DMSO-*d*, using tetramethylsilane as an internal reference. XRD profiles were measured with a Rigaku D/max 2400 diffractometer with Cu KR radiation. Gel permeation chromatography (GPC) analysis was performed on a Malvern instrument connected to one refractive index detector (Viscotek-VE3580-RI-DETECTOR), using a DMF solution containing 1 g·L^−1^ of lithium bromide as solvent at a flow rate of 0.8 mL·min^−1^ at 55 °C, and calibrated with polystyrene standards. Thermogravimetric analysis (TGA) was conducted with a PerkinElmer Pyris 6 TGA. Experiments were carried out on approximately 6–8 mg powder samples heated in flowing nitrogen (flow rate = 20 cm^3^·min^−1^) at a heating rate of 10 °C·min^−1^. The morphology observation of the samples was carried out on atom force microscopy (AFM, Nanoscope IIIa digital instrument, VECCO Co.) equipped with a silicon cantilever (typical spring constant 40 N m^−1^) in tapping mode under ambient conditions. UV-vis absorption spectra were recorded using a SHIMADZU UV-1700 spectrophotometer whereas the photoluminescence solution spectra were registered on a Jasco FP-6200 spectrometer with Xenon lamp as the light source. Cyclic voltammetry (CV) measurements were conducted on a CHI 660E electrochemical workstation at a scan rate of 50 mV·s^−1^ with a 0.1 mol·L^−1^ solution of LiClO_4_ as an electrolyte in dry acetonitrile (CH_3_CN). Density-functional theory (DFT) calculations were performed using the B3LYP functional as implemented in Gaussian 03 program. Spectroelectrochemical experiments were used to evaluate the optical properties of the electrochromic films. For the investigations, the cured films were cast on an ITO-coated glass slide, and a homemade electrochemical cell was built from a commercial UV-visible cuvette. The cell was placed in the optical path of the sample light beam in a UV-vis-NIR spectrophotometer, which allowed us to acquire electronic absorption spectra under potential control in a 0.1 mol·L^−1^ LiClO_4_/CH_3_CN solution.

### Data availability statement

The authors declare that all the data are true and available.

## Electronic supplementary material

Supplementary information
