# Supplementary material for: Organic-inorganic hybrid electrochromic materials, polysilsesquioxanes containing triarylamine, changing color from colorless to blue
Source: Sci Rep. 2017 Nov 7;7:14627. doi: 10.1038/s41598-017-15337-1 (PMC5677139; doi:10.1038/s41598-017-15337-1)
Supplement: Supplementary file 1 — Supplementary information [file 41598_2017_15337_MOESM1_ESM.pdf]

Supplementary information

Organic-inorganic hybrid electrochromic materials, polysilsesquioxanes containing triarylamine, changing color from colorless to blue

Shuzhong Wang[a], Shuwei Cai[a], Wanan Cai[a], Haijun Niu[a*], Cheng Wang[a], Xuduo Bai[a], Wen Wang[b*], Yanjun Hou[a].

[a] Key Laboratory of Functional Inorganic Material Chemistry, Ministry of Education, Department of Macromolecular Science and Engineering, School of Chemical, Chemical Engineering and Materials, Heilongjiang University, Harbin 150086, P R China

[b] School of Materials Science and Engineering, Harbin Institute of Technology, Harbin 150080, P R China

*corresponding author. Email: haijunniu@hotmail.com; wangwen@hit.edu.cn

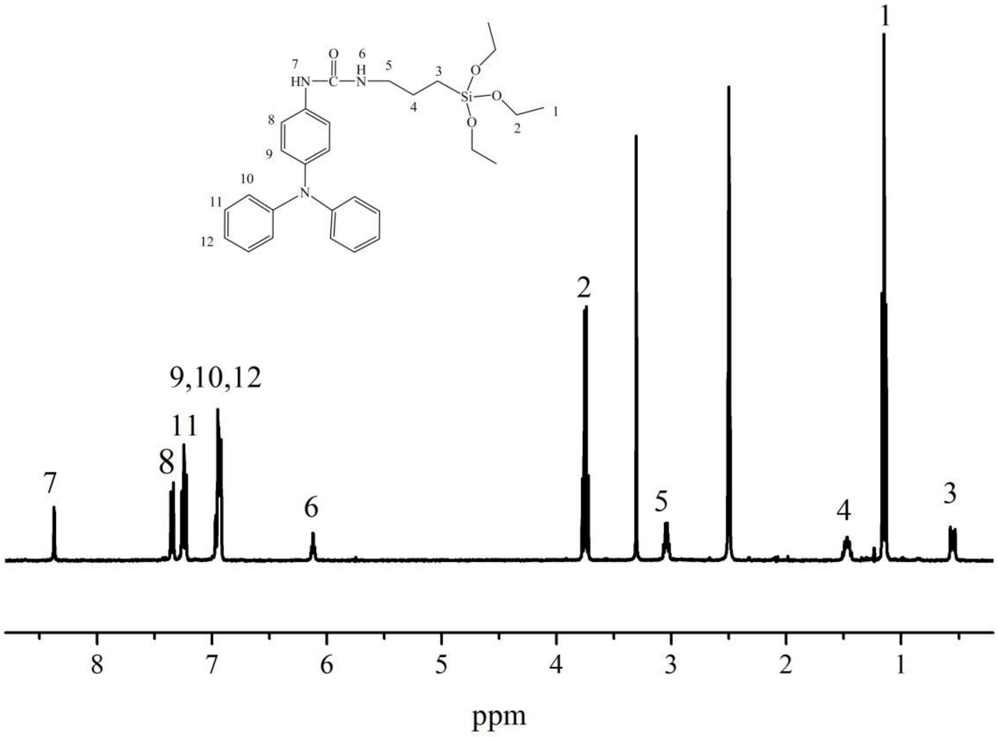

Figure S1. ^1H NMR spectra of DPAP-TEOSPU.

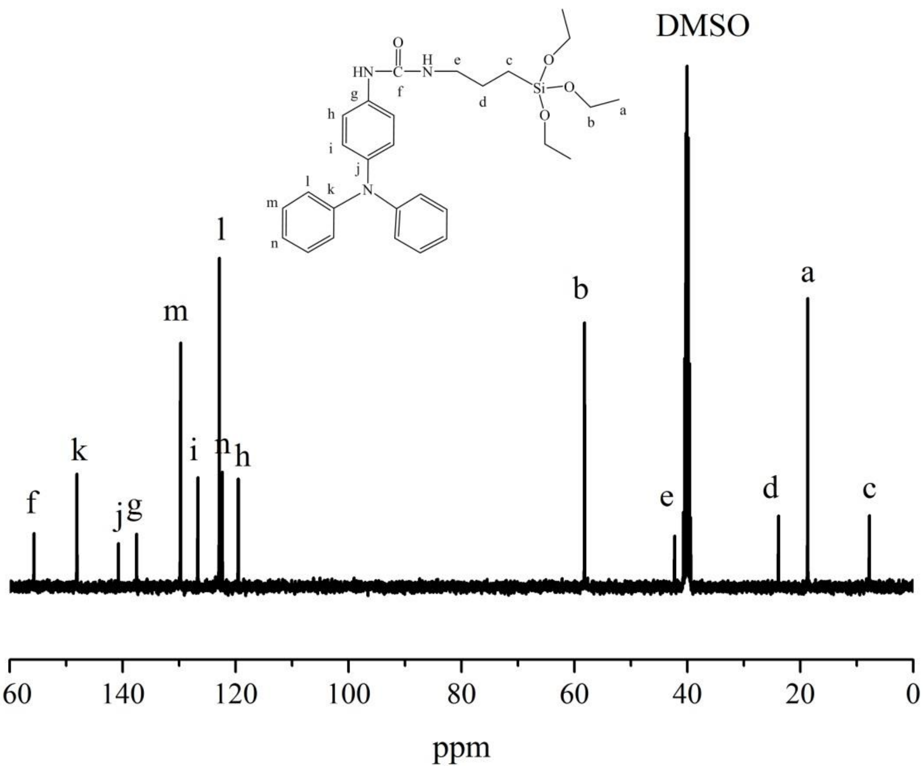

Figure S2. ^{13}C NMR spectra of DPAP-TEOSPU.

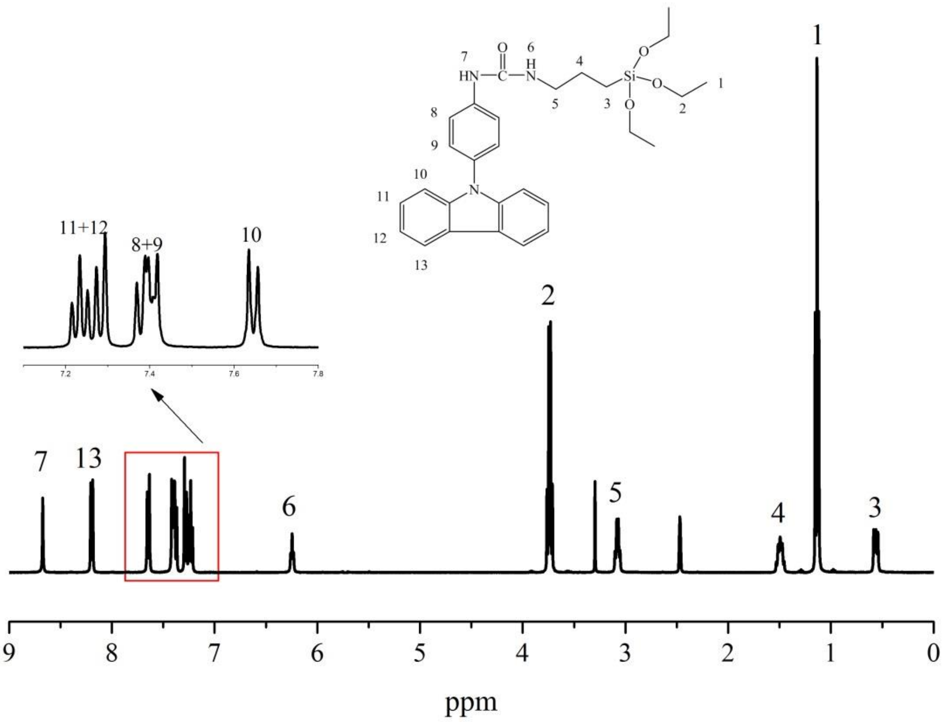

Figure S3. ^{1}H NMR spectra of CzP-TEOSPU.

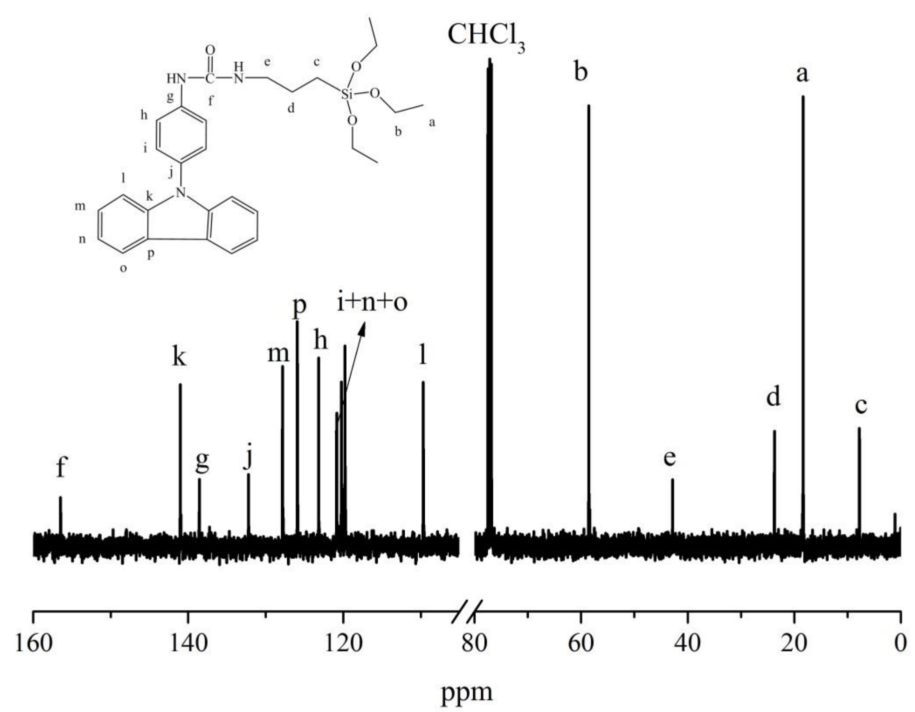

Figure S4. ^{13}C NMR spectra of CzP-TEOSPU.

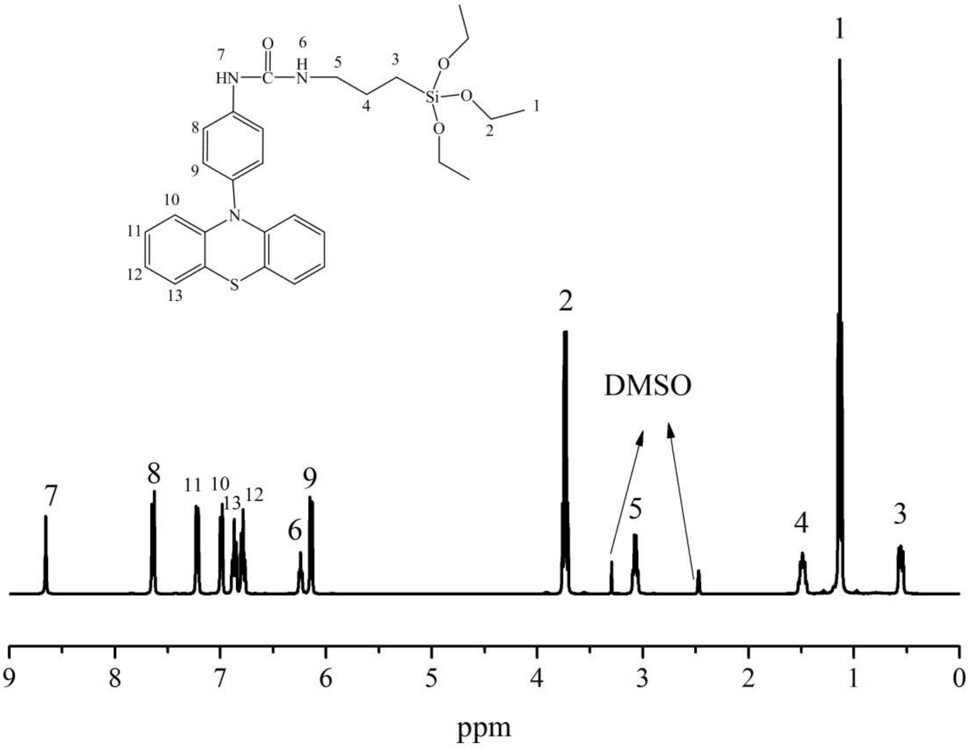

Figure S5. ^{1}H NMR spectra of PTP-TEOSPU.

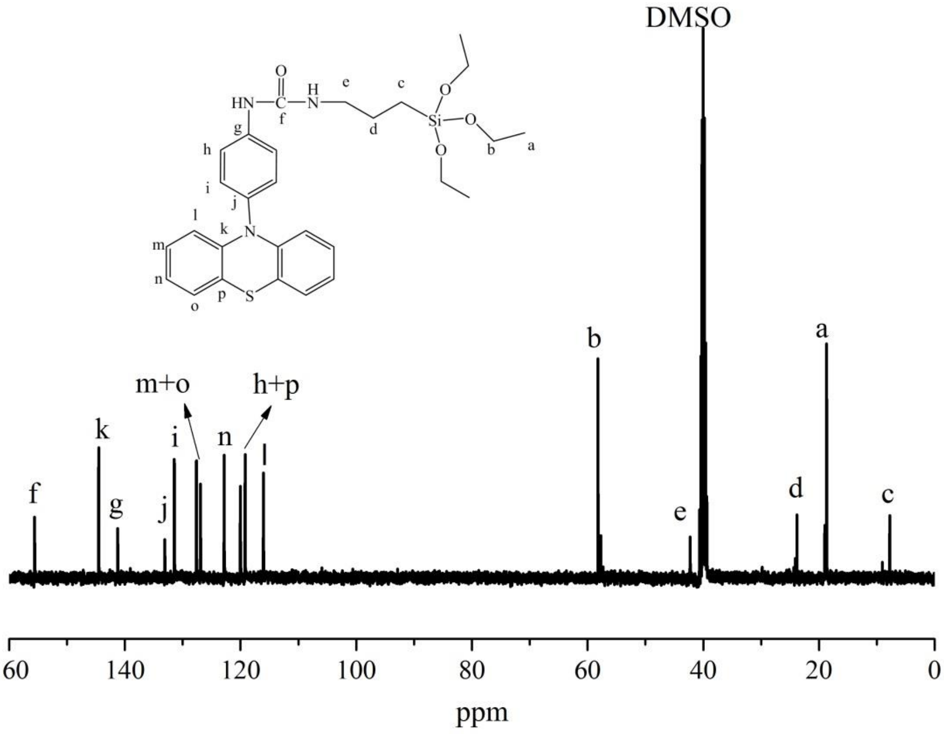

Figure S6. ^{13}C NMR spectra of PTP-TEOSPU.

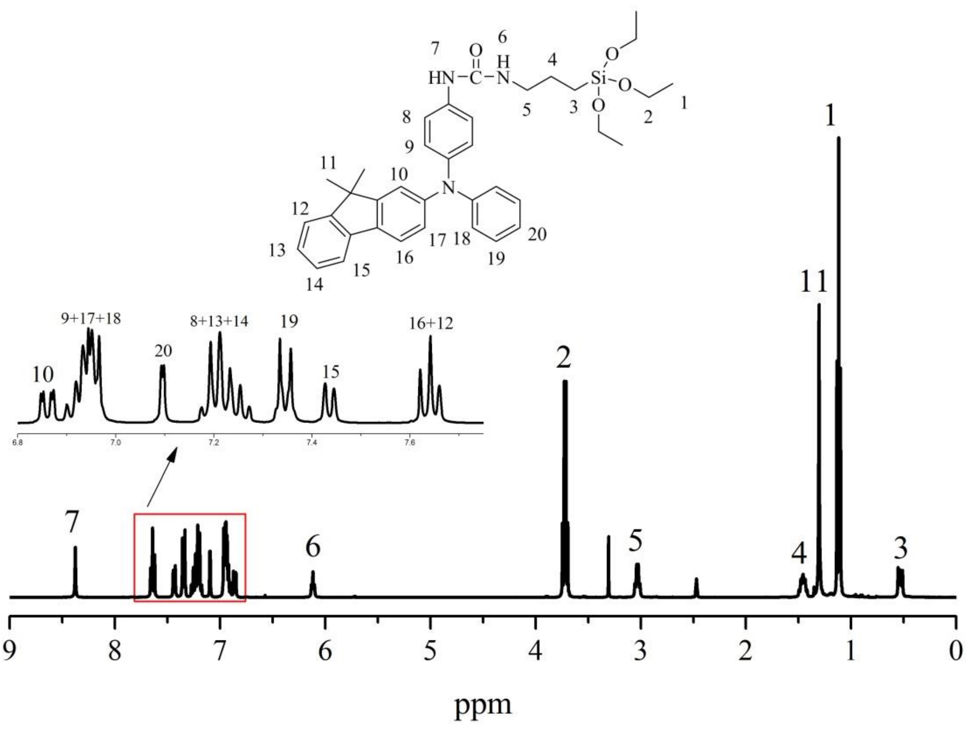

Figure S7. ^{1}H NMR spectra of DFPAP-TEOSPU.

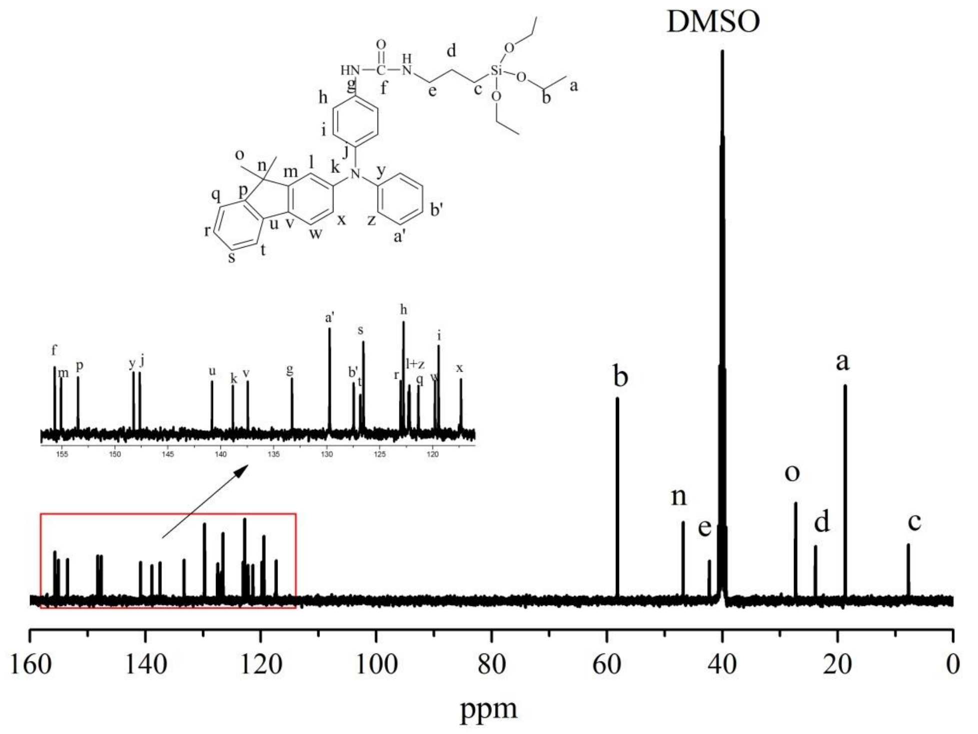

Figure S8. ^{13}C NMR spectra of DFPAP-TEOSPU.

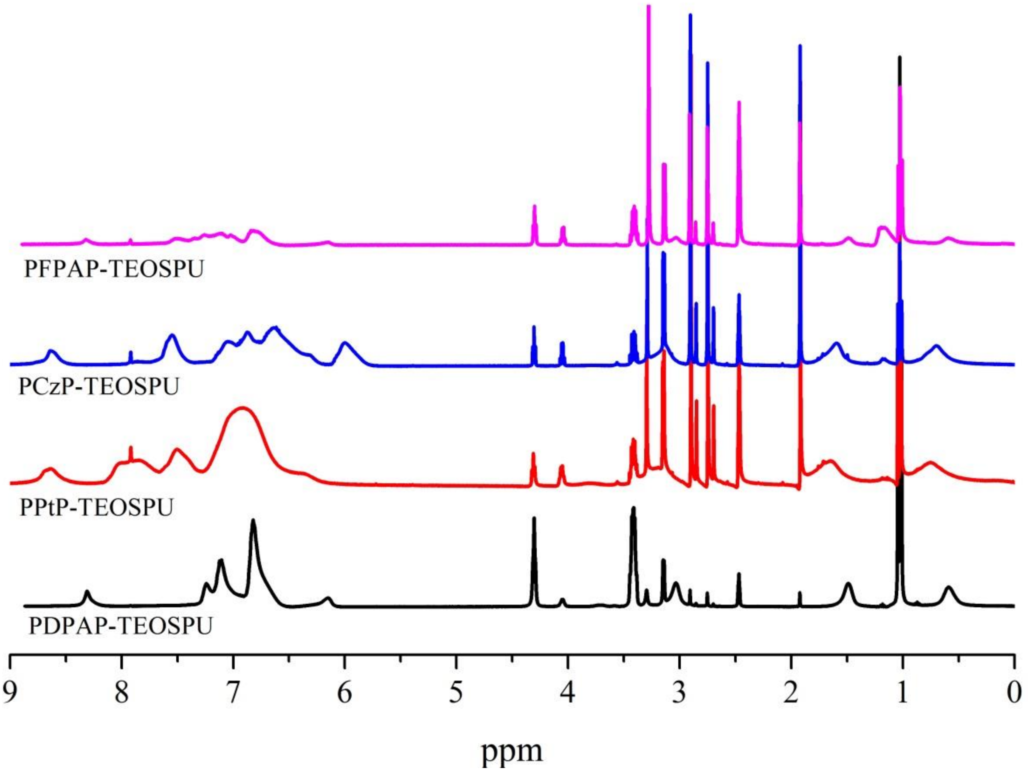

Figure S9. ^{1}H NMR spectra of polymers.

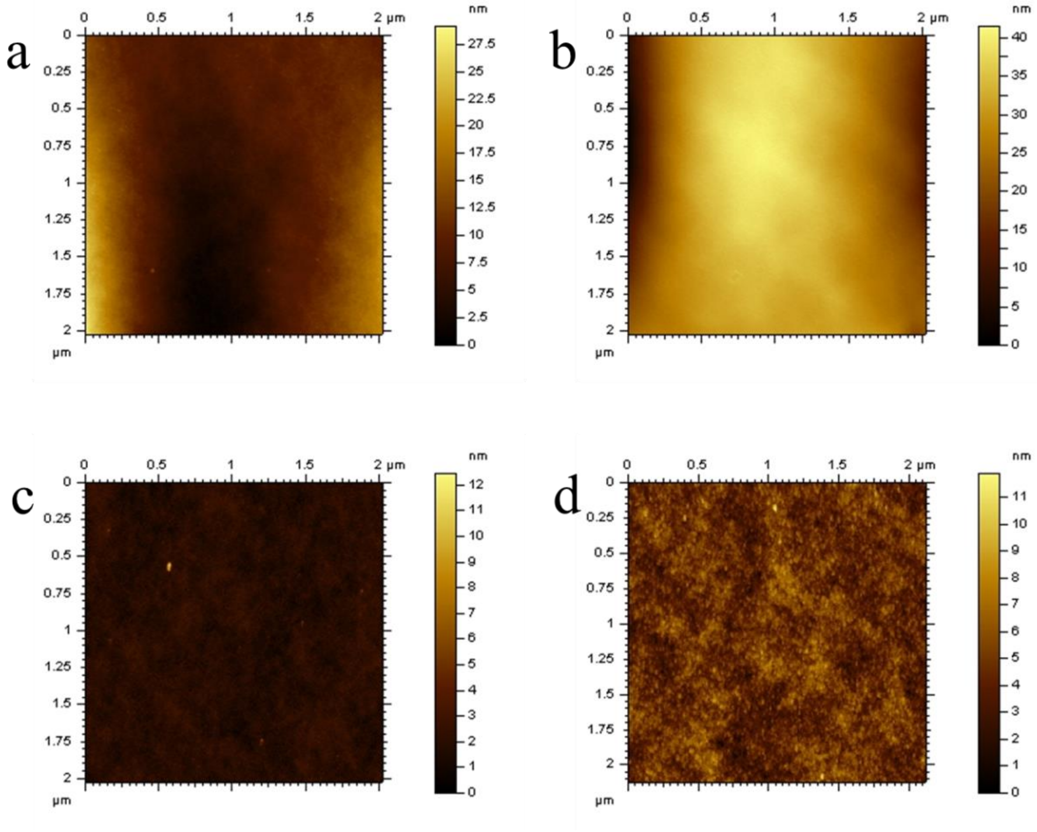

Figure S10. AFM images of a, b for PDPAP-TEOSPU c, d for PPTP-TEOSPU before and after CV experiment in tapping mode.

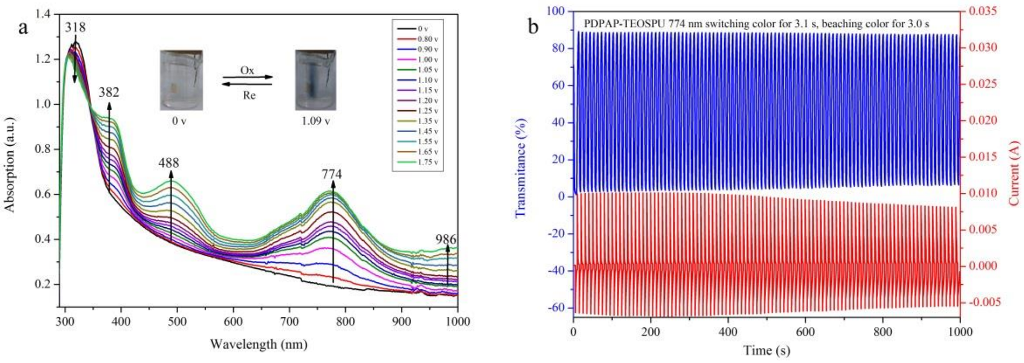

Figure S11. Electronic absorption spectra of PDPAP-TEOSPU thin film (a) and dynamic changes of the transmittance and current (b).

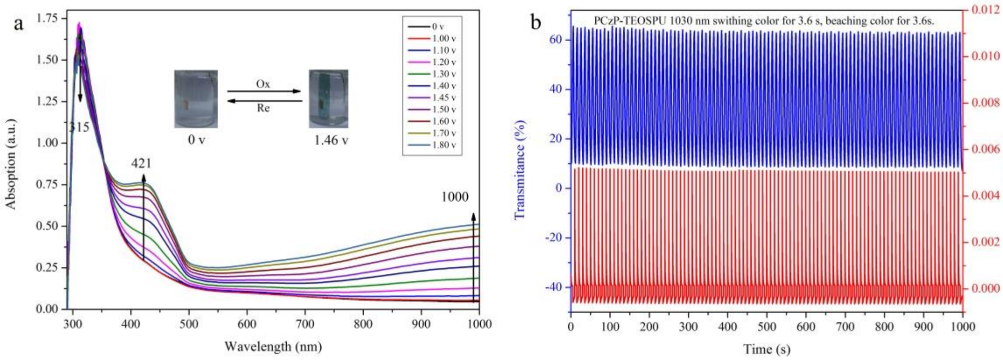

Figure S12. Electronic absorption spectra of PCzP-TEOSPU thin film (a) and dynamic changes of the transmittance and current (b).

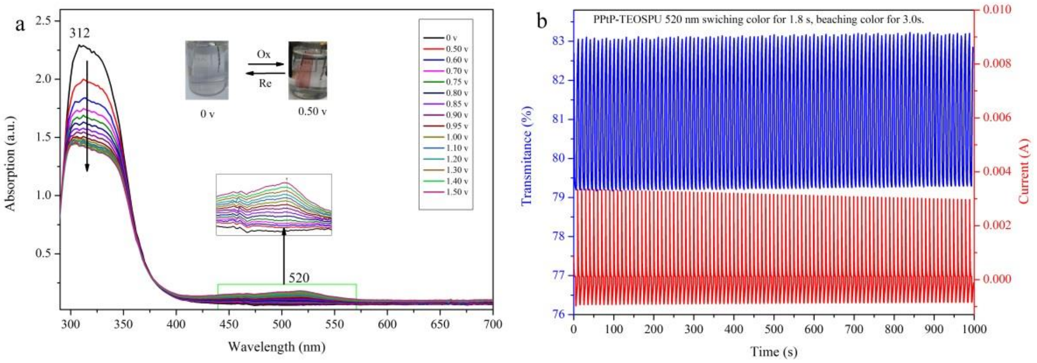

Figure S13. Electronic absorption spectra of PPTP-TEOSPU thin film (a) and dynamic changes of the transmittance and current (b).

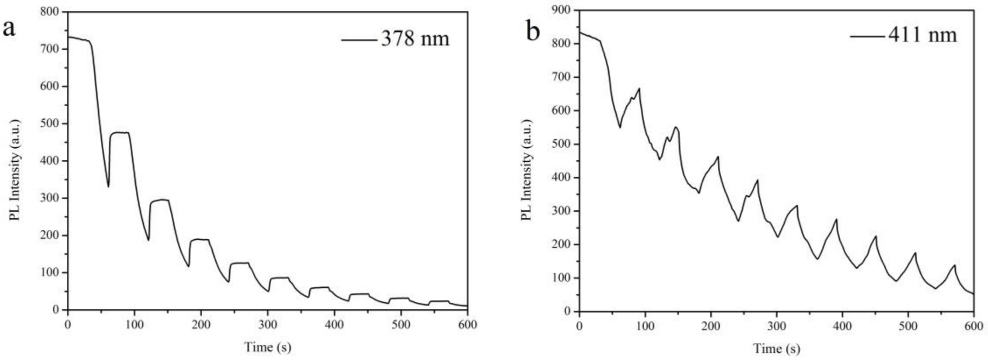

Figure S14. Fluorescence switching response of PCzP-TEOSPU (a) and PDFPAP-TEOSPU (b) under applied step potential between 0 V and 1.5 V with a cycle time of 60 s.

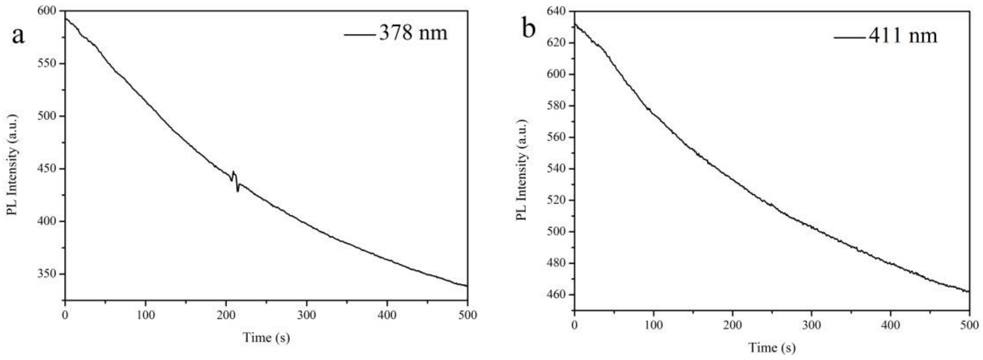

Figure S15. Changes in PL spectra of PCzP-TEOSPU (a) and PDFPAP-TEOSPU (b) with no potential applied.

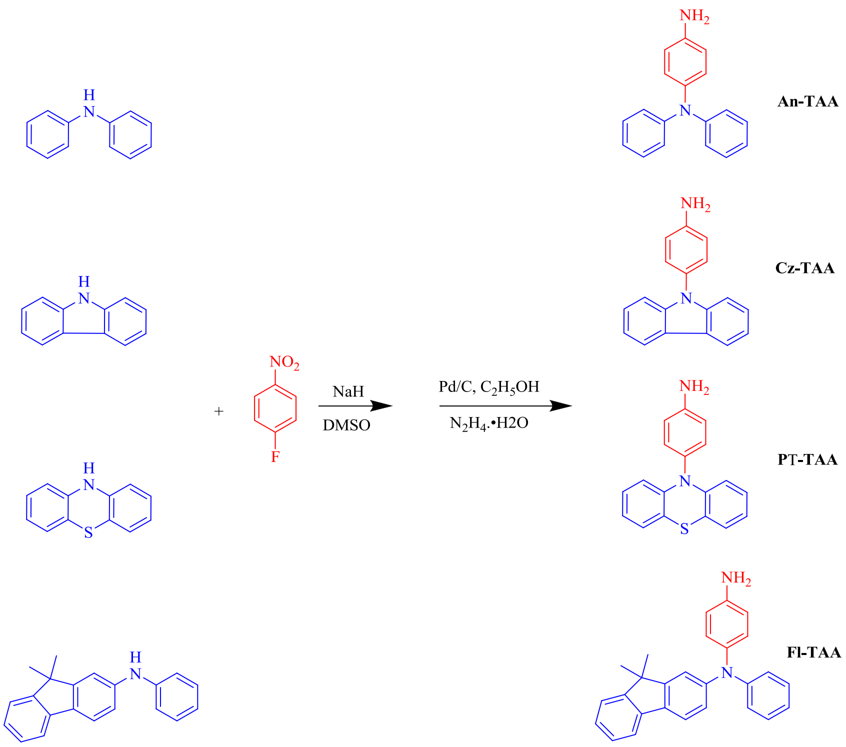

Figure S16. Synthesis routes of TAA derivatives.
